# Progress in Diseases of the Blood

**Published:** 1903-06-27

**Authors:** 


### Progress in Diseases of the Blood.

**Blood Examination.**—The Johns Hopkins Hospital Bulletin[1] publishes a series of results obtained by different observers using the various cytometers and hæmoglobinometers, to show what variations may be introduced in each by the personal equation, and what degree of agreement exists between the readings of different kinds of instruments. Between the Thoma-Zeiss and Oliver's instruments the greatest discrepancy is 500,000; the average of 17 counts is 190,000 cells per cm. These readings are made with nearly normal bloods. The point which Oliver made, that his invention is not accurate for the anæmias, is emphasised. In pernicious anæmia it gives results which are too high. In chlorotic states of the blood its reading is below the true "count." Another point which is brought out is the necessity of using freshly made acetic acid solution when diluting the blood in the Thoma-Zeiss "white" pipette: yeast-torulæ, which are very like lymphocytes, grow in dilute acetic acid. To secure the freshness of the fluid a bottle is used to which, when full of stained water, a given number of drops of glacial acetic acid is added. The hæmocrit is dismissed shortly. As a means of detecting lipæmia, cholæmia, or hæmoglobinæmia, it is useful, but not as a cytometer. As to hæmoglobinometers, "it is much to be desired that soon no instruments will read in percentages, but all in grams per 100 c.c. of blood." Different makers take different standards for normal when graduating their tubes, and the 13-14 grams per 100 c.c. standard usually employed gives for adults a normal of only 87 per cent., while a quite normal child's blood would read only 75–80 per cent. The Dare hæmoglobinometer is recommended. Different observers with separate instruments agree markedly in their results when testing the same blood; and it is notorious that two persons will disagree about the matching of colour in other instruments.

**Pernicious Anæmia.**—W. Hunter[2] regards this as a definite disease, with characteristic lesions and a definite cause. In every one of 25 cases he has found a special form of glossitis, and, associated with this, diseased conditions of the stomach and intestines, "septic gastritis," gastric atrophy, follicular enlargement in the intestine, congestion and catarrh of the intestine, and slight patchy colitis. There was also in 24 out of the 25 cases preceding or existing "oral sepsis." This oral sepsis is the preparation of the ground for a specific hæmolytic infection which produces the special lesion of the tongue, and the Streptococcus longus was found in the tongue, blood, and spleen in his more recent cases on which he made a bacteriological examination. The tongue lesion is characterised by the formation of little vesicles, often accompanied by desquamation and the formation of small ulcers. At times there is swelling and tenderness of the tongue. Tenderness was noticed about the time of onset of the anæmia in every case. It is generally worse at first, and subsides later, and as the disease goes on the tongue often gets to look clean and harmless. But the lesion invariably recurs if the case is watched for long, though "the degree of anæmia may interfere with the occurrence of marked inflammatory changes." The final result is atrophy of the mucosa, leaving a smooth, glossy surface.

**Banti's Disease—Operation.**—Benedetto Schiassi[3] reports a case of this disease which he first treated by paracentesis; then, the cirrhosis of the liver being advanced, he implanted the omentum in the abdominal muscles. This not proving sufficient to prevent the recurrence of ascites, he grafted the spleen on the parietal peritoneum. After six months the patient was in a greatly improved condition in every respect, the ascites having reappeared in small quantity only, soon after the operation, and when this was drawn off there was no re-accumulation. The functions of the liver were improved by the relief to the portal vessels afforded by the new vascular channels.

1 Johns Hopkins Bulletin, Jan., 1903. 2 Clin. Jour., Oct. 29. 3 Med. Rec., Nov. 22.

(*To be continued.*)

